# What shall we call God? An exploration of metaphors coded from descriptions of God from a large U.S. undergraduate sample

**DOI:** 10.1371/journal.pone.0254626

**Published:** 2021-07-12

**Authors:** Adam K. Fetterman, Nicholas D. Evans, Julie J. Exline, Brian P. Meier

**Affiliations:** 1 Department of Psychology, University of Houston, Houston, Texas, United States of America; 2 Department of Psychology, University of Texas at El Paso, El Paso, Texas, United States of America; 3 Department of Psychology, Case Western Reserve University, Cleveland, Ohio, United States of America; 4 Department of Psychology, Gettysburg College, Gettysburg, Pennsylvania, United States of America; Coventry University, UNITED KINGDOM

## Abstract

People use numerous metaphors to describe God. God is seen as a bearded man, light, and love. Based on metaphor theories, the metaphors people use to refer to God reflect how people think about God and could, in turn, reflect their worldview. However, little work has explored the common metaphors for God. This was the purpose of the current investigation. Four trained raters coded open-ended responses from predominantly Christian U.S. undergraduates (N = 2,923) describing God for the presence or absence of numerous metaphoric categories. We then assessed the frequency of each of the metaphor categories. We identified 16 metaphor categories that were present in more than 1% of the responses. The top categories were “GOD IS POWER,” “GOD IS HUMAN,” and “GOD IS MALE.” These findings were similar across religious affiliations. We attempted to support our coding analysis using top-down and bottom-up automated language analysis. Results from these analyses provided added confidence to our conclusions. We discuss the implications of our findings and the potential for future studies investigating important psychological and behavioral outcomes of using different metaphors for God.

## Introduction

God is conceptualized in many ways, even within religions. Religion researchers have spent considerable time exploring these conceptualizations and the outcomes of individual differences in their endorsement. Most of this work has focused on the character of God. Researchers reason that if we know a person’s conceptualization of God’s character, then we can predict important psychological outcomes, and ample evidence supports this idea. Researchers have adopted various methods to extract or measure conceptualizations of God. However, little work has looked at the common metaphors or metonymies used to conceptualize God. This lack of work is important because metaphor and metonymy are quite relevant for these conceptual purposes [1]. Therefore, we performed a systematic analysis, using multiple methods, to identify the common metaphors and metonymies used to describe God in a large sample of predominantly Christian college students. Due to the combination of the Christian background of our participants and our own experiences in the Judeo-Christian United States, we will primarily draw from these traditions in our theorizing.

### Conceptualizing God

There are many ways people conceptualize God. One key insight involves the self. For example, Epley et al. [2] found strong correlations between what people believe and what they think God believes. Therefore, it seems that God may be created in our image, rather than vice versa. That is, one strong conceptualization of God is based on a reflection of one’s self.

Prior work has also focused on various personality characteristics of God. Common characteristics analyzed in the literature focus on anthropomorphic God concepts [3], benevolent versus authoritarian God concepts [4], and controlling God concepts [5], to name a few. Of course, there are also God concepts directly derived from religious traditions, like the Holy Trinity [6]. However, probably one of the most systematic approaches to identifying conceptualizations of God is the work of Johnson et al. [7]. They created a five-factor model of God concepts. These factors include authoritarian, benevolent, limitless, mystical, and ineffable. Overall, there are many conceptualizations of God, and Johnson et al. [7] provide a good overview of these while developing their model.

Johnson et al. [7] focused on the most frequent adjectives that people use to describe God’s nature. However, we only know of one study that has sought to systematically uncover the common metaphors for God; what God *is*. In this case, DesCamp and Sweetser [8] analyzed the common metaphoric source domains used to describe God in a number of holy books (described in context below). Our goal, here, was to explore common metaphors for God as they are revealed through people’s descriptions. Of course, any conceptualization of God *is* metaphoric, because, theologically, God is omnipresent and omniscient (i.e., God simply “is”), at least in most Abrahamic traditions. Even so, humans are limited in their ability to comprehend such non-natural entities and must conceptualize God within their physical boundaries [9]. Specifically, humans are limited in that they cannot think past their bodies and experiences and, therefore, liken God to things based on their physical experiences [10, 11]. This is likely where metaphoric processes begin.

### Defining and identifying metaphors for God

Metaphors consist of a “source” and a “target” [12]. The “target” is a to-be-described concept that is relatively abstract, while the “source” is a referent used to describe what the “target” is like and is relatively concrete in meaning. This source is either paired with the target (metaphor: “The lawyer is a shark”) or used in place of the target (metonymy: “Going to see the shark”). In other words, a seemingly unrelated, but relatively more concrete concept is used to understand a relatively more abstract or intangible concept. This is the premise of metaphor theories [1, 12–14]. Importantly, people leverage metaphors because they are useful, not because they beautify our language, as we are taught in high school English [15]. They serve an epistemic or understanding function [16] and present as a type of trace evidence of people’s thought processes regarding complex and intangible ideas [17]. Given that God is intangible by definition [9], and many people spend their lives trying to understand and find meaning through their ideas about God [18], metaphors should be particularly useful for this purpose. In turn, identifying metaphoric source categories for God should be insightful for understanding how people conceptualize God.

Of course, people do not offer up metaphors for God in such a clear SOURCE-TARGET mapping (e.g., GOD IS HUMAN). They mostly provide descriptors and images. However, these descriptors and images can provide key insights into the metaphoric conceptualizations of God. For example, if people use human-like descriptors for God, we can infer that they understand the concept of God through their existing knowledge about humans. This maps well with Barrett and Keil’s [9] theory of anthropomorphism. The anthropomorphizing process is metaphoric, because people are using a relatively concrete referent (i.e., human) to think about an abstract concept (i.e., God). That is, people may not *literally* think God is human, but they use humanness (the source) to ground or concretize the God concept (the target). This is the purpose of metaphors as they are studied in the broader literature [14].

In sum, we define metaphor, in line with the broader metaphor literature, as the non-literal use of a source domain to refer to a relatively abstract concept [1, 12–14]. We can assume, based on the “unknowable” nature of God held by most theologies [9], that most descriptions of a God are metaphoric—using something knowable to understand the unknowable. Therefore, we set out to infer the most common metaphoric source categories from open-ended descriptions of God (the target domain).

### Metaphoric God concepts

Given the bodily and experiential limits on human cognition, the easiest way to think metaphorically about a supernatural entity, like God, is by giving it human-like characteristics (i.e., anthropomorphizing). Indeed, the imagery of God as a human is apparent in art and media (e.g., The Sistine Chapel, statues, the television show *The Simpsons* or the film *Dogma*) and such images are instantiations of metaphoric processes. The way people report their relationships with God is similar to their relationships with other humans [19], and they often bestow God with human-like agency and experiences [3]. As such, the primary metaphoric source category for God is likely a human-like conceptualization, even though this is theologically inaccurate [9]. Supporting this, Meier and Fetterman [20] found an implicit metaphoric association between God-related words and human words (vs. non-human words). Further, DesCamp and Sweetser [8] found that “human with agency” was one of the most common source categories in religious texts and Kunkel et al. [21] found similar results in a small sample of God images. In the current investigation, we expected a ubiquitous “GOD IS HUMAN” metaphor or metonymy.

In addition to using human-based metaphors, people commonly refer to God using male pronouns, at least in Christian traditions. In fact, it is difficult to refer to God without using “he.” This is even true with common depictions of God, once again (e.g., the bearded man in the sky). Further, at least in Christian religions, God is referred to as “Father” (e.g., Psalms 89:26, “He shall cry unto me, Thou art my Father, My God, and the rock of my salvation”). Even in Judaism, while God is not considered gendered, the Hebrew Bible uses the masculine gender form. DesCamp and Sweetser [8] also point to the commonality of the male gender in religious texts. Therefore, we expected a common metaphoric or metonymic theme of “GOD IS MALE.”

Beyond these more human-like conceptualizations of God, religious teachings provide additional source categories for God metaphors. Nearly every religion depicts God as the “CREATOR,” or by using “POWER” terms. For example, note Jeremiah 32:17: “Ah Lord GOD! Behold, Thou hast made the heavens and the earth by Thy great power and by Thine outstretched arm! Nothing is too difficult for Thee.” Of course, describing God as “powerful” is barely metaphorical. It is yet another adjective to describe God’s attributes. Even so, there seems to be a consistent theme of metaphors or, in this case, metonymies that would fall under a “GOD IS POWER” category. For example, God is THE Creator (e.g., John 1:3 states, “All things were made through him, and without him was not anything made that was made.”). Further, many Gods are depicted as forces of power or nature (e.g., Thor with lightning and thunder). DesCamp and Sweetser [8] further note the common use of the “king” as a metaphoric source category in religious texts. We should also note that “power” is not a particularly concrete source concept. However, the concepts of “authority,” “creator,” and “force” have imagery that may allow people to conceptualize the idea of omniscience or omnipotence, which leads to metonymical expressions like “The Creator.” Overall, “GOD IS POWER” is likely to be a common metaphoric or metonymic category.

The human, male, and power metaphoric source categories are likely to be most common, especially if our results follow the religious texts [8] of our predominantly Christian sample, but people also discuss God using relatively less frequent metaphorical or metonymical source categories. As such, we expected three more source categories to be relatively frequent. First, religious teachings and expressions refer to God as love (e.g., in John 4:8, “Anyone who does not love does not know God, because God is love”). Second, God is depicted in vertical space (e.g., Psalm 57:2, “I cry out to God Most High, to God who fulfills his purpose for me”). Indeed, prior research has shown a metaphoric association between God and higher vertical space [3, 19, 20]. Finally, some consider God or “God” as the natural world or the cosmos (e.g., a form of pantheism; [22]). As such, one might expect less frequent, but nonetheless common, metaphorical or metonymic categories of “GOD IS LOVE,” “GOD IS UP,” and “GOD IS THE COSMOS/NATURAL ORDER.”

In sum, there are numerous ways to conceptualize God metaphorically. Our ultimate goal, here, was to identify the most common metaphors and metonymies used to refer to God using a methodological triangulation approach [23]. Specifically, we used a combination of language coding, top-down automated language analysis, and bottom-up automated language analysis, each method requiring qualitative interpretation—i.e., to fit the descriptions into metaphoric source categories—in its own right. We have proposed six source categories (“HUMAN,” “MALE,” “POWER,” “LOVE,” “UP,” and “COSMOS/NATURAL ORDER”) that seem apparent from common imagery used to depict and linguistically refer to God. However, we intended to use a data-driven approach, which allowed natural language to help us derive as many metaphorical and metonymical source categories as were commonly used, in a relative sense. Therefore, our investigation is necessarily exploratory in nature.

## Materials and methods

### Participants

We collected data from 3,071 undergraduates attending three universities in the United States. Participants completed a large questionnaire on their religious beliefs and demographics and described God. Of the original sample, 2,923 (1,826 female, 1,085 male, 12 unreported; *M*_age_ = 19.10, *SD*_age_ = 1.80; *Min*_age_ = 18, *Max*_age_ = 51) participants responded to the focal item after providing demographic information, including their religious affiliation. The study was approved by the Case Western University Institutional Review Board. Informed consent was in written form.

The original data collection was carried out to examine separate research questions by the third author. The current investigation focused on a single open-ended question (“Please try to give a brief description of what the term God means to you in the space below”). We were also interested in briefly exploring the consistencies between these responses based on religious affiliation (frequencies are in Table 2). Coded data are available at https://osf.io/rpzav/.

### Coding procedures

To code the God description responses for metaphoric source categories, we recruited four raters (2 Ph.D. students and 2 undergraduate students). Following the recommendations of Fetterman et al. [24] and the Metaphor Identification Procedure [25], we first explained the concept of metaphors and metaphor theory to the raters and ensured that they understood. The group then developed a code-book with instructions and a list of metaphoric source categories used to describe God based upon religious writings and common beliefs (See Table 1 for a full list of the categories and examples from each category). The instructions included tips for coding, such as coding for what God is and not what God does; to not overthink the coding; to note that some responses may fit more than one category; to not allow one’s personal beliefs to influence ratings; and to create a list of difficult-to-interpret responses to discuss during the weekly coding meetings. After receiving the instructions, the raters separately coded the first 10 responses. We then met as a group to discuss and reconcile discrepancies in this initial coding. The remaining coding task commenced, and regular meetings occurred (with waning frequency) until all raters were finished. During the meetings, the raters were allowed to propose additional categories that came up. No additional categories were added.

**Table 1:** Code categories, examples, interrater reliability, and percentage of the sample that used each metaphoric source category.

Code Categories	Instructional Examples	Gwet’s AC1	% Frequency
Power	omniscience, creator, almighty, ominpotent	.82	60.07
Human	mother, father	.88	56.72
Male	he, father, son, his	.95	50.94
Concrete entity	figure, tangible being	.39	36.20
Supernatural/divine	ghost, spirit, angel	.88	17.00
Up	high, up, higher, above	.98	16.97
Emotion	love, compassion, goodness	.93	09.78
Love	love	.98	08.55
Support	protector, helper	.92	07.83
Cognitive Process	knowledge	.91	05.75
Cosmos/Universe	space, everything, stars	.97	04.27
Collective	friend, humanity	.98	03.08
Natural Process	Big Bang, evolution	.99	02.26
Journey	path, plan	.99	01.51
Place	heaven, space, dimensions	.98	01.09
Earth	plants, trees, dirt	.99	01.06
Self	"I/we are all god"	.99	00.79
Female	she, mother, hers	> .99	00.55
Light	illumination, sun	> .99	00.48
Body/organ	brain, heart	> .99	00.48
Soul	soul	> .99	00.34
Sun	sunshine	> .99	00.10
Non-human Animal	beasts, mythical creatures	> .99	00.03
Sensation	warmth, feeling	> .99	00.03
Air	breath, clouds, sky	> .99	00.00

Overall, the raters read the response and then decided whether each word in the description was being used metaphorically. If it was, they located the category and gave it a “1,” indicating that the category was present. The categories that were not present received a “0.” For example, if a participant wrote, “He is the universe,” the coders might have given the response a “1” for the male category and a “1” for the cosmos category.

To ensure that one rater did not have too much influence over the coding results, we gave the source category a “1” if more than one rater applied a code to any particular response. If only one rater applied the code, we gave the response a “0” for that source category. Therefore, each response could have a “1” (present) or “0” (absent) for each source category.

### Automated language analysis procedures

The second approach we used to identify metaphoric source categories was top-down automated language analysis. Specifically, we used the Linguistic Inquiry and Word Count (LIWC) software [26]. LIWC automatically quantifies the percentage of words used from preset categories (e.g., “power” or “affect”) in a writing sample. Each of the categories represents a theme, into which various words can fall (i.e., top-down). For example, the category “affect” contains words such as “happy” and “cried.” Every time LIWC detects a word from a category, it is tallied toward a total score for that language category. The category score is then divided by the total word count for the sample to get an average score.

We put all responses into a single file and analyzed it as a whole, instead of analyzing each participant’s response separately because writing samples were short and uneven in length. Analyzing the responses separately would result in language categories being over-weighted. For example, if one participant’s response is simply “power,” the power category receives a score of 100%. Compare that to the participant who writes “God is power.” For this person, the power category receives ~33%. However, qualitatively, the participants have written the same thing. As such, compiling all responses into one file allows us to avoid undue influence by word count.

While automated language analyses are not optimal for identifying metaphors for psychological purposes [24], in combination with qualitative interpretation, it may provide indirect insight into general themes of the responses. This, indirectly, could further inform what source categories people are thinking about when processing the God concept. For example, if people are using a lot of words that fall into the “positive emotion” category of the LIWC, it may be that they are thinking about God in terms of positive experiences, like love.

The third approach we used to identify metaphoric source categories was a word frequency analysis using word clouds. This is a more bottom-up approach to automated language analysis, relative to the LIWC. To do so, we used the Meaning Extraction Helper (MEH; [27]). Specifically, we used its frequency output feature to prepare the data for our word cloud generation. In this case, the MEH reads all the text responses and outputs a raw count of each of the words used, minus “stop words.” Stop words are the most typical words used in everyday language (e.g., “the”). In addition to the preset stop words, we included “God,” as nearly every participant used this word to describe God (e.g., “God is…”). What is left is an output that lists the words and the number of times they were used across all the responses. We then entered this data into R and used the “wordcloud2” package to create a word cloud. The largest words in a word cloud are the most frequent words and the smallest are the least frequent words. Again, the qualitative interpretation of this word frequency analysis enhanced our confidence in the interpretation of the results from the prior methods.

## Results

### Coding results

The results from the metaphor source category coding procedure are shown in Tables 1 and 2. First, to assess interrater agreement, we calculated Gwet’s AC1 [28] using R and the “gwet.ac1.raw” function in the “irrCAC” package. We chose Gwet’s AC1 over the more traditional Fleiss’ Kappa [29] given that it is a more stable method for estimating interrater reliability [30]. Further, Fleiss’ Kappa can be influenced by the prevalence of a code and return low coefficients in low prevalence cases even when agreement is nearly perfect [28]. Gwet’s AC1 does not suffer from this bias. For example, in our own data, instances in the “GOD IS AIR” category were less than 1% in frequency. The interrater reliability in this category had a Fleiss’ Kappa of .20 and a Gwet’s AC1 of .998. In contrast, for the high frequency (i.e., 56%) category “GOD IS HUMAN,” the coefficients were identical (i.e., .88). Overall, all source categories had high interrater reliability according to Gwet’s AC1 (see Table 1). The lowest was for “GOD IS A CONCRETE ENTITY,” but it was still acceptable.

**Table 2:** Percentage of the sample that used each metaphoric source category by religion and in total, in order of frequency from left (most) to right (least).

		%Power		%Human		%Male		%Concrete Entity		%Supernatural		%Up		%Emotion		%Love		%Support		%Cognitive Process		%Cosmos		%Collective		%Natural Process		%Journey		%Place		%Earth	
Religious Category	*N*	*M*	*SD*	*M*	*SD*	*M*	*SD*	*M*	*SD*	*M*	*SD*	*M*	*SD*	*M*	*SD*	*M*	*SD*	*M*	*SD*	*M*	*SD*	*M*	*SD*	*M*	*SD*	*M*	*SD*	*M*	*SD*	*M*	*SD*	*M*	*SD*
Catholic	611	58.76	49.27	56.14	49.66	49.10	50.03	34.21	47.48	14.57	35.31	19.80	39.88	12.11	32.65	7.69	26.67	6.38	24.47	4.42	20.57	3.76	19.05	2.62	15.98	1.15	10.65	1.31	11.38	0.65	8.07	0.16	4.05
Protestant	603	70.48	45.65	68.99	46.29	64.84	47.79	35.99	48.04	19.57	39.71	15.59	36.31	18.91	39.19	13.43	34.13	8.79	28.34	2.65	16.09	3.65	18.76	2.99	17.03	0.83	9.08	1.16	10.72	0.50	7.04	0.33	5.75
Christian	975	68.51	46.47	72.21	44.82	68.41	46.51	32.00	46.67	18.77	39.07	9.95	29.95	21.03	40.77	16.62	37.24	11.69	32.15	1.64	12.71	4.62	20.99	3.69	18.87	0.72	8.45	1.64	12.71	1.85	13.47	1.23	11.03
Jewish	42	54.76	50.38	19.05	39.74	16.67	37.72	35.71	48.50	14.29	35.42	33.33	47.71	7.14	26.07	0.00	0.00	4.76	21.55	9.52	29.71	4.76	21.55	2.38	15.43	0.00	0.00	0.00	0.00	0.00	0.00	2.38	15.43
Muslim	21	57.14	50.71	52.38	51.18	47.62	51.18	52.38	51.18	9.52	30.08	19.05	40.24	14.29	35.86	4.76	21.82	9.52	30.08	0.00	0.00	9.52	30.08	0.00	0.00	0.00	0.00	4.76	21.82	0.00	0.00	0.00	0.00
Hindu	37	64.86	48.40	29.73	46.34	16.22	37.37	51.35	50.67	21.62	41.73	29.73	46.34	8.11	27.67	5.41	22.92	0.00	0.00	8.11	27.67	8.11	27.67	8.11	27.67	10.81	31.48	0.00	0.00	0.00	0.00	0.00	0.00
Buddhist	15	46.67	51.64	33.33	48.80	20.00	41.40	53.33	51.64	33.33	48.80	20.00	41.40	6.67	25.82	6.67	25.82	0.00	0.00	26.67	45.77	0.00	0.00	6.67	25.82	13.33	35.19	0.00	0.00	0.00	0.00	6.67	25.82
Spiritual	13	30.77	48.04	30.77	48.04	30.77	48.04	38.46	50.64	0.00	0.00	46.15	51.89	23.08	43.85	23.08	43.85	0.00	0.00	15.38	37.55	23.08	43.85	7.69	27.74	30.77	48.04	0.00	0.00	0.00	0.00	0.00	0.00
Atheist	119	36.13	48.24	13.45	34.26	9.24	29.09	44.54	49.91	18.49	38.98	13.45	34.26	4.20	20.15	0.84	9.17	3.36	18.10	30.25	46.13	4.20	20.15	0.84	9.17	8.40	27.86	0.84	9.17	0.84	9.17	1.68	12.91
Agnostic	166	46.99	50.06	18.67	39.09	13.86	34.65	46.99	50.06	13.25	34.01	35.54	48.01	6.63	24.95	0.60	7.76	2.41	15.38	12.65	33.34	4.82	21.48	2.41	15.38	9.64	29.60	1.20	10.94	1.20	10.94	1.81	13.36
None	210	36.67	48.30	29.05	45.51	13.81	34.58	41.90	49.46	13.33	34.07	21.43	41.13	4.76	21.35	1.90	13.70	2.86	16.70	16.19	36.92	0.95	9.74	1.90	13.70	3.81	19.19	3.33	17.99	0.48	6.90	1.43	11.90
Other	29	37.93	49.38	27.59	45.49	20.69	41.23	34.48	48.37	6.90	25.79	24.14	43.55	3.45	18.57	3.45	18.57	0.00	0.00	10.34	30.99	17.24	38.44	13.79	35.09	6.90	25.79	3.45	18.57	0.00	0.00	10.34	30.99
Eastern Orthodox	19	52.63	51.30	47.37	51.30	36.84	49.56	42.11	50.73	36.84	49.56	21.05	41.89	21.05	41.89	21.05	41.89	5.26	22.94	0.00	0.00	10.53	31.53	0.00	0.00	0.00	0.00	5.26	22.94	0.00	0.00	0.00	0.00
Unsure	9	44.44	52.70	55.56	52.70	33.33	50.00	33.33	50.00	0.00	0.00	44.44	52.70	11.11	33.33	0.00	0.00	0.00	0.00	0.00	0.00	0.00	0.00	0.00	0.00	0.00	0.00	0.00	0.00	0.00	0.00	0.00	0.00
No Response	54	51.85	50.43	48.15	50.43	40.74	49.60	40.74	49.60	9.26	29.26	20.37	40.65	7.41	26.44	5.56	23.12	7.41	26.44	3.70	19.06	5.56	23.12	1.85	13.61	1.85	13.61	0.00	0.00	5.56	23.12	5.56	23.12
TOTAL	2923	60.66	48.86	56.72	49.55	50.94	50.00	36.20	48.06	17.00	37.57	16.97	37.54	15.12	35.83	10.64	30.84	7.83	26.88	5.75	23.28	4.28	20.24	3.08	17.28	2.26	14.86	1.51	12.18	1.09	10.41	1.06	10.25

The frequencies of the source categories are in Table 1. In Table 2, we have broken these source category frequencies down by religious affiliation for categories that were present in more than 1% of the responses. Overall, the most prevalent metaphor source category was “GOD IS POWER.” Again, this is somewhat counter-intuitive, because power itself is an abstract concept. However, power imagery is likely used most often because God is seen as having ultimate control over everything (i.e., omniscient and omnipresent) and this is hard for humans to imagine [9]. Therefore, when there is nothing else to explain a phenomenon, an unknowable “force” or “creator” is at play. This focus on “power” may also explain some of the imagery in various religions (e.g., Thor and thunder) and depictions of various Gods as being extremely powerful. Results also suggest that the use of “power” metaphors is most prevalent in those with “Christian” religious affiliations and least prevalent for those who identified as “spiritual.” Even so, the “GOD IS POWER” source category was consistently high for all religious affiliations.

The next two most common metaphor source categories were “GOD IS HUMAN” and “GOD IS MALE.” Though their prevalence is not equal, they likely came together (e.g., “father” and “son” are coded as both male and human). These two themes being highly prevalent make sense and fit our general predictions. When people are picturing God, they are likely seeing some sort of human and male figure, based on their embodied cognitive limitations [10, 11] and iconic imagery. These findings also reflect depictions of God (e.g., the Sistine Chapel) and reveal a concrete figure that has an agency that people can understand with relative ease [3, 8, 9, 21]. These categories were most prevalent in those who identified as Protestant or generically Christian. They were least prevalent among those who identify as atheist and agnostic. This also makes sense, as a “GOD IS MALE/HUMAN” represents something tangible and real, whereas these groups have lower belief in a “real” or physical God. Interestingly, Jewish participants also used these source categories less. This fits with prior work showing that Jewish participants in the United States tend to score high on Johnson et al.’s [7] mystical or effable categories [31].

The next most common source category was “GOD IS A CONCRETE ENTITY.” This was a catch-all category for descriptions of “some physical being” that was not necessarily human. This theme was relatively less common for Christians of all sorts, including Catholics. It was more common for Muslim and Buddhist participants, compared to the other affiliations.

After the concrete entity source category, there was a dramatic drop in prevalence. The next most prevalent source category, “GOD IS SUPERNATURAL,” was less than half as prevalent as the prior one. However, those who identified as Eastern Orthodox and Buddhist used this category more frequently than the other affiliations. For the former, this appears to be driven by the common mention of a “spirit,” and for the latter, “divine” was a frequently used word.

The remaining source categories were relatively less frequent in all Abrahamic faiths, with one notable exception. For Jewish participants, “GOD IS UP” was relatively common. This might be because they used fewer human source categories and the next most prevalent source category was “up.” The same can be said for the prevalence of “GOD IS UP” for those who identified as spiritual and those who responded “unsure” to the religious affiliation question.

Those who identified as spiritual also used source categories of “GOD IS EMOTION,” “GOD IS LOVE,” “GOD IS THE COSMOS,” and “GOD IS NATURAL PROCESS” with relatively more frequency than the other affiliations. However, there were relatively few of these participants and they indicated that they just believe in “something.” Atheists used “GOD IS COGNITIVE PROCESS” sources, which was driven by their descriptions of God as “an idea.”

Overall, we identified 16 metaphorical source categories that people use when describing God at a frequency of at least 1% or higher. The most common were power-related and human-like in their imagery and this somewhat transcends religious affiliation. These source categories will be useful for understanding how people conceptualize God and differences in these conceptualizations will be informative for understanding psychological and behavioral outcomes.

### Language analysis results

#### LIWC

**Fig 1:**
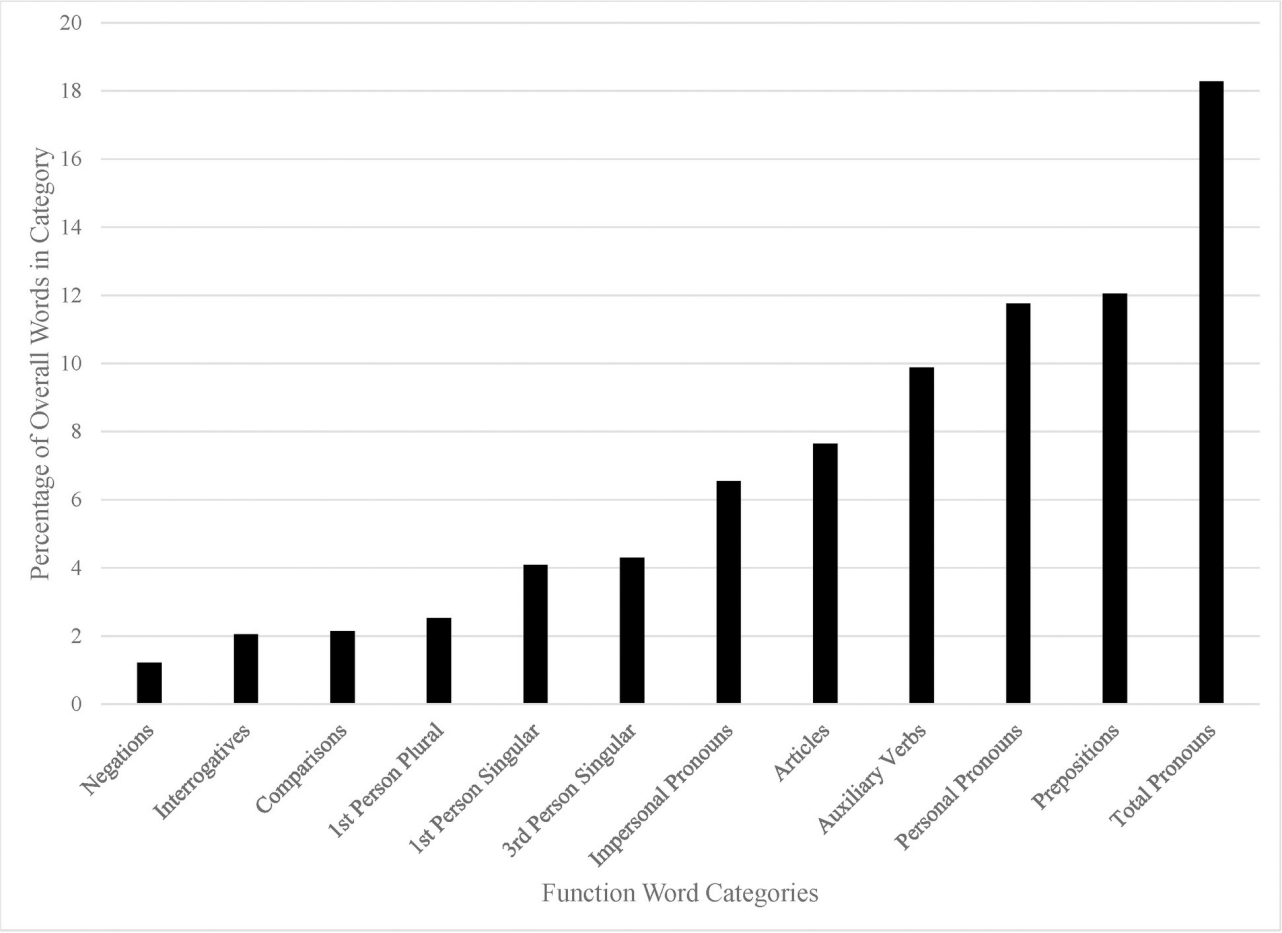
Percentage of word frequency for each of the LIWC function word categories.

**Fig 2:**
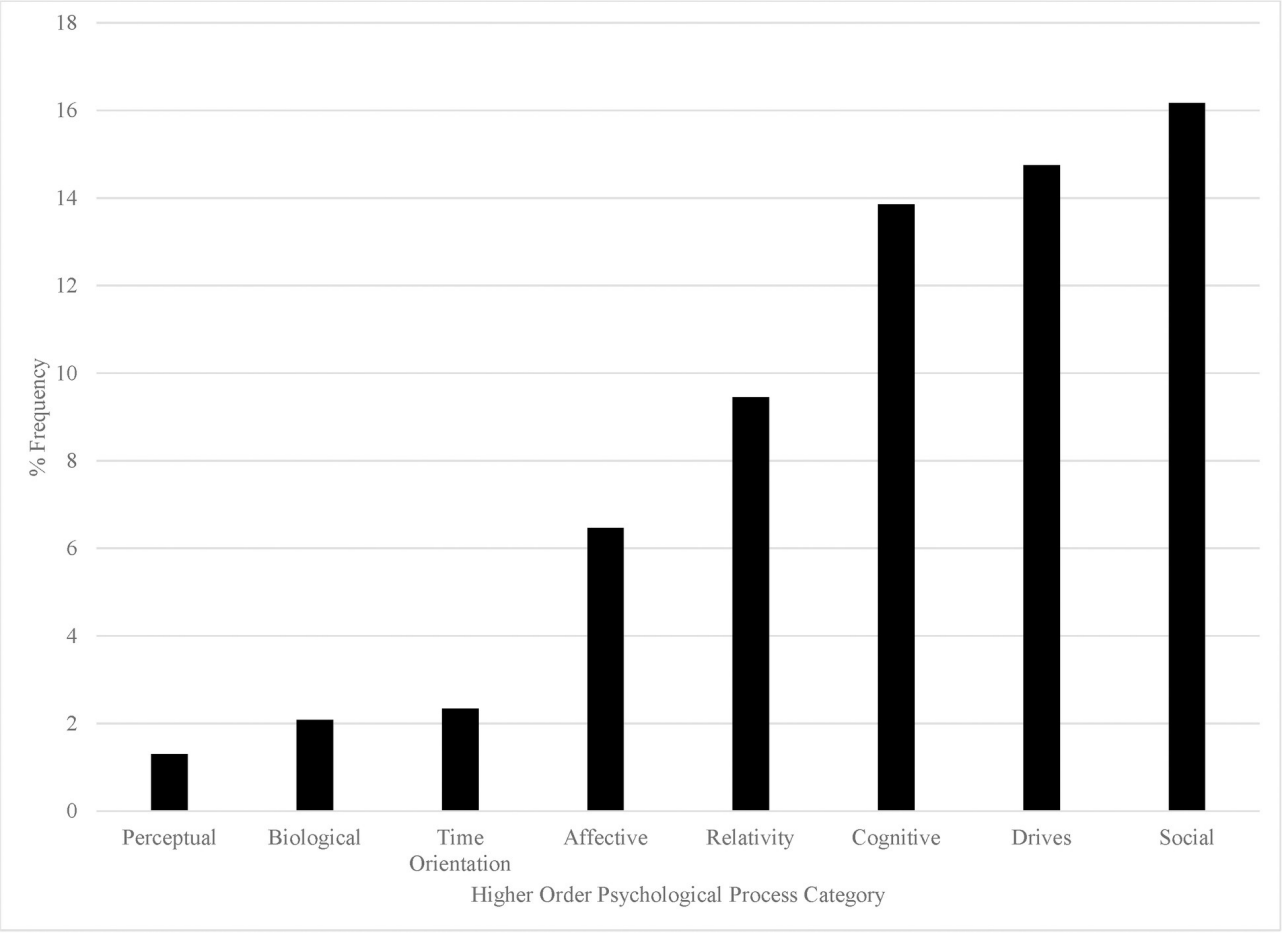
Percentage of word frequency for each of the LIWC higher order psychological process categories.

**Fig 3:**
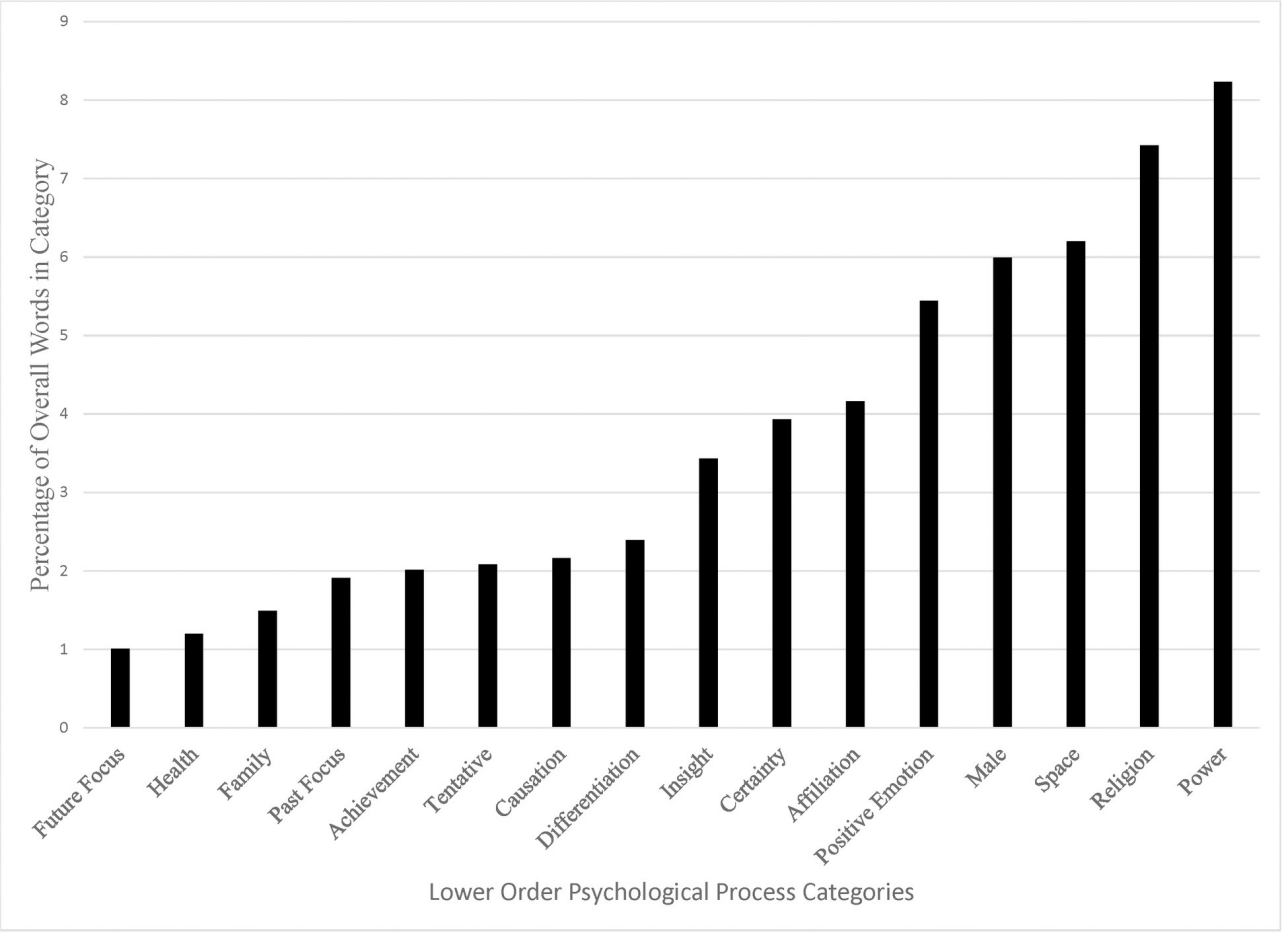
Percentage of word frequency for each of the LIWC lower order psychological process categories.

The results of the language analysis using the LIWC 2015 dictionary are in Figs 1–3. Although we analyzed all LIWC 2015 categories (for further description see the LIWC2015 manual [26], which is freely available online), we only present language categories that readily inform our research question. Namely, we present those which contain words that would be used to (e.g., “love” or “power), or in the process of (e.g., “he” or “it”), describing the God concept. Other categories (e.g., “money” or “netspeak”) were off-topic. The numbers on the y-axis represent the percentage of words present in the overall sample from each category, relative to the total word count. We also only present categories that were higher than 1% in frequency. Below, we describe the most notable findings from these analyses.

In Fig 1, we have plotted the frequency of common linguistic dimensions. The most frequent category of linguistic dimensions was pronouns (which includes all pronoun types). This reflects the commonality of references to God as “he” or “it.” It also supports our “GOD IS HUMAN,” “GOD IS MALE,” and “GOD IS A CONCRETE ENTITY” metaphoric source category findings in the coding section. Clearly, participants were thinking of an agentic being. The next two categories are prepositions and personal pronouns (e.g., “he”). Again, the latter supports our coding findings. It should be noted that impersonal pronouns (e.g., “it”), which would fit the “concrete entity” theme, is a little more than half as frequent as personal pronouns, meaning people seem to implicitly think of God as human and male, even if they do not explicitly endorse the idea that God is human. The prepositions category reflects the common use of temporal and spatial modifiers of God. It would seem that in describing what God is, people are often describing where God is (e.g., “up” or “above” or “in”). Another interesting frequent category is auxiliary verbs, which seem to indicate that participants described actions that God has done (perfect tense) or does (do-support). These may not represent a metaphoric category but instead gives another hint toward the conceptualization of God as an agentic being.

In Fig 2, we have plotted the frequency of the higher-order psychological process language categories present in the language used to describe God. These higher-order categories are broader ones, which can be further separated by low-order categories (e.g, the drives category includes lower-order categories like power, affiliation, achievement, etc.). The most frequent higher-order category was social processes. The fact that “he” and “father” are coded in this category likely explains its high frequency. The second most frequent category was psychological drives. Both of these findings fit with our coding results. The cognitive processes category was nearly as frequent as the drives. This category includes words related to causation, certainty, and insight. This, once again, likely reflects the common theme of power we found in our coding, as “powerful” things “affect” things with finality. Next was the relativity and affective process categories. The former likely reflects the orientational state of God (e.g., “up” or “higher”). The latter reflects the frequency of emotion-related (e.g., “love”) words that appeared somewhat frequently in our coding.

In Fig 3, we have plotted the frequent lower-order psychological process categories. The most frequent word category was power. Again, this replicated our coding results. God was commonly conceptualized as an omnipotent being that can influence the happenings of the world. The next three most frequent categories were space, male, and positive emotion. This supports our coding and interpretation of the higher-order category findings. Common metaphorical themes are related to God as a male entity that is located up or in a higher dimension and is related to positive emotional experiences, like love.

In all, the findings of the common linguistic dimensions and the higher- and lower-order psychological processes from the LIWC analysis support our coding results. While this method requires more qualitative interpretation, it was worthwhile to examine how automatic language might reflect our coding [24]. God appears to be conceptualized as an omnipotent concrete entity that many consider a “father” and is associated with a higher orientation and positive emotional states.

#### Word frequency analysis (word cloud)

**Fig 4:**
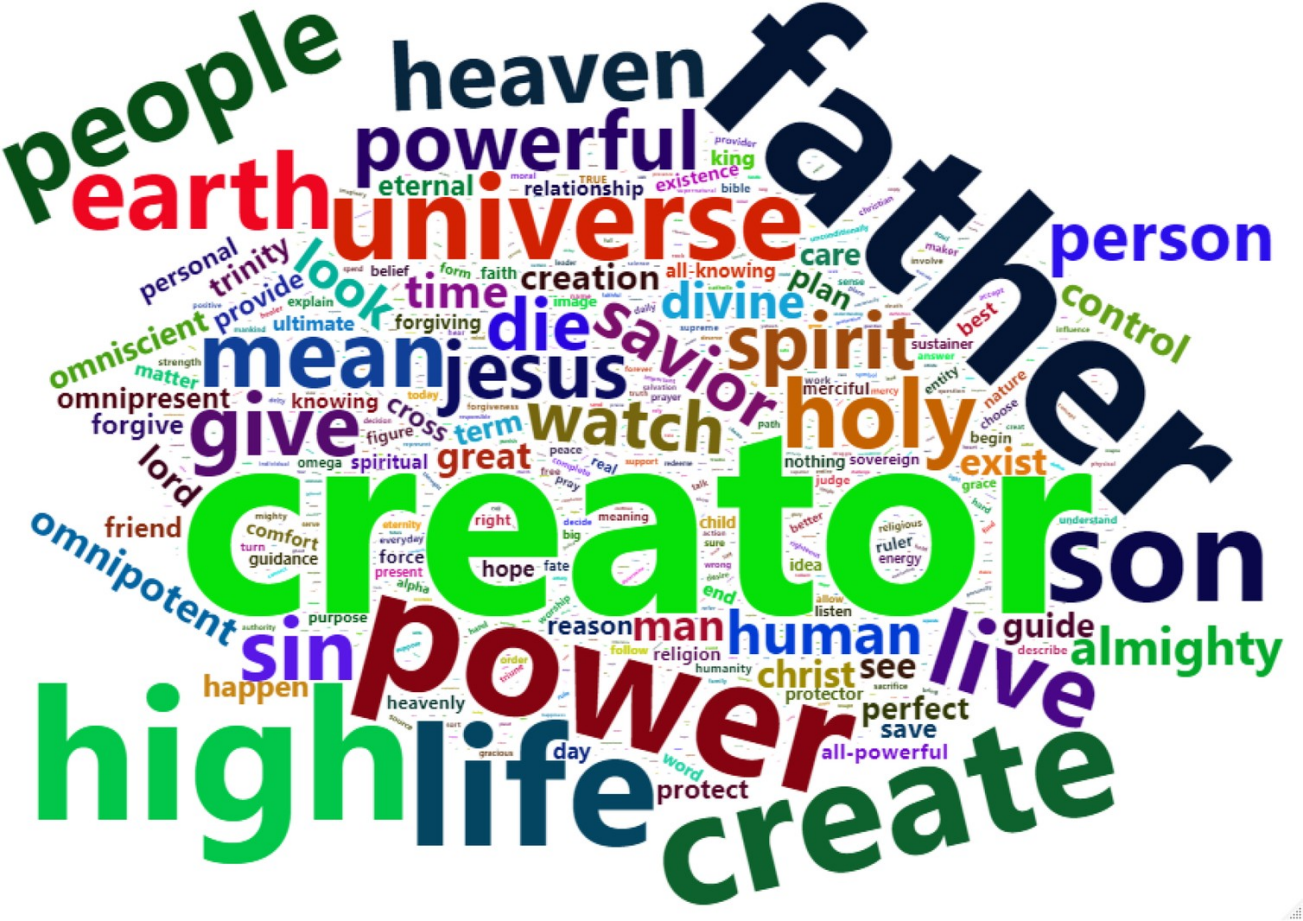
Word cloud of raw word frequencies with larger words being more frequent and smaller words less frequent.

In Fig 4, we present a word cloud to display the most frequently used words to describe God. The largest and most frequent word was “Creator.” This fits with our coding results, as “creator” is inherently powerful and is a concrete entity. Indeed, we see that the word “power” was relatively large as well. Another large word was “Father,” which tracks with our coding results and the maleness of the God concept. Interestingly, the word “love” appeared to be relatively frequent as well. While our metaphorical source category “GOD IS LOVE” was present in around 10% of the descriptions in our coding results, the word frequency analysis might suggest that it was a more frequent theme than we anticipated. However, an alternative account might be that participants were using this word to describe what God does (e.g., “God loves us”), not what God is (e.g., “God is love”), which would account for the discrepancy. A perusal of the responses confirms this alternative explanation. Finally, “high” is another frequently used word. This fits with the frequency of “GOD IS UP” source categories in the coding results.

## Discussion

We often read and hear God being described in numerous metaphors and metonymies (e.g., a bearded man, creator, up, and love). These metaphors represent how people understand God, and perhaps the world. However, a systematic examination of the actual metaphors used in people’s descriptions of God does not exist. Therefore, we sought to identify the most commonly used metaphoric source categories for God. To do so, we analyzed thousands of open-ended descriptions of God, using hand-coding procedures as well as top-down and bottom-up automated language analyses. The results were consistent across methods and with an analysis of religious texts [8]. People most commonly used power and human imagery when thinking about God. Other source categories included maleness, vertical space, and love. In total, we identified 16 source categories that were used at least 1% of the time in our large sample. This was the first important step in (1) understanding what metaphors people use to describe God and (2) laying the groundwork for determining how the metaphors people use to discuss God relate to important psychological and behavioral outcomes.

### Implications

Our investigation is informative for numerous literatures related to religious cognition. Particularly, the results support prior research and provide numerous avenues for future work on God conceptualization and the outcomes of such conceptualizations. Furthermore, the current work informs coding and analysis procedures when applied to a unique topic like God. We discuss each of these implications and corresponding future directions related to them, in turn.

The results of the current investigation support prior work on the conceptualization of God. Particularly, the frequency of power imagery reflects DesCamp and Sweetser’s [8] analysis of religious texts, Kunkel et al.’s [21] analysis of a small sample of God images, and Johnson et al.’s [7] “limitless” and “authoritarian” factors. Further, it may be related to the controlling God concept investigated by Landau et al. [5]. From this standpoint, future work should investigate correlations between the use of “GOD IS POWER” metaphors and these factors. Additionally, Landau et al. [5] found that belief in a controlling God was associated with an increased belief that God intervenes in the world. Therefore, we predict that using power-related source categories to refer to God will be related to such interventionist beliefs.

Nearly all prior work on God conceptualizations has an anthropomorphic theme [3, 8, 9, 21]. Accordingly, our finding on the frequency of “GOD IS HUMAN” metaphors is not surprising. It is nearly impossible to imagine something outside our own bodily experience and this applies to God, as well [10, 11]. Of course, which type of human-like figure God inhabits may vary widely. Here, we found that God is referred to in male terms, likely due to our heavily Christian sample. This matches recent work by Jackson et al. [32], which found that people tend to think God looks like an average-looking smiling young man. In fact, in our data, even those participants who suggested God has no gender referred to God as “he.” In this sense, one might consider the “he” as a pronoun stand-in or generic language feature with little meaning. However, from both a metaphoric [33] and general [34] social cognition perspective, the use of “he” likely brings with it a schema-wide network of associations that can impact thoughts, feelings, and even behavior. In fact, recent work found that conceptualizing God as a white male led to racial and gender discrimination in the workplace [35]. This suggests that the pronoun “he” is consequential.

Broadly, future work should investigate how people who are more likely to use “GOD IS HUMAN” metaphors differ compared to those who are less likely to do so. For example, human source domain endorsement might be positively associated with authoritarian/benevolent God beliefs. Further, it might be positively associated with religious behavior, such as praying to God for decision-making advice, as one would seek with a parent, and attachment to God [19]. We see strong possibilities for research investigating important psychological and behavioral outcomes associated with these individual differences.

The “GOD IS HUMAN” metaphor is highly concrete, which likely explains its ubiquity. However, some of the more frequent categories were less concrete. For example, “GOD IS LOVE” is the mapping of an abstract concept (“God”) with another relatively abstract concept (“love”). On the other hand, love is *relatively* more concrete than God for some. If God is ineffable, love is at least a little more effable. People have multiple physical and emotional experiences they associate with love that they can then use to ground the God concept. Even so, because the purpose of metaphors is to provide meaning to relatively abstract concepts by making them more concrete [12], less concrete metaphors likely provide less meaning to abstract concepts. A recent study found that using a concrete metaphor for life increased meaning in life, for example [36]. Therefore, more abstract or less concrete source categories may not provide as much meaning to God.

Consequently, if people are trying to find the meaning of God, or meaning in life through God [18], then using a relatively less concrete metaphoric source may lead to relatively less meaning derivation. More specifically, if a person seeks meaning through God, but uses less concrete source domains, then they may fail to find meaning through God. This failure could also be associated alienation and depression (e.g., [37]). Of course, it could also be the case that someone who endorses “GOD IS LOVE,” for example, finds meaning elsewhere (e.g., relationships with family) or uses multiple metaphors. Future work is needed to disentangle such considerations.

### Methodological considerations and limitations

In addition to supporting prior work and inspiring new lines of research, our procedures provide some insights for future explorations of God metaphors and metonymies. Here, we used a triangulation approach [23] involving hand-coding procedures based on the Metaphor Identification Procedure [25], automated top-down language analysis, and bottom-up word frequency analysis (i.e., word clouds). Each method has its benefits and limitations [24] and some are unique to the current task: identifying metaphors for God. We focus on the ones unique to the metaphors for God task.

Using the coding procedures to identify metaphors for God proved challenging. Specifically, it required us to train raters to focus on descriptions of what God is, rather than what God does or God’s personality. This differs from other metaphor identification studies in that those studies tend to focus on the conceptualization of non-agentic target domains, like an orgasm [38] or tourism [39]. They do not have an agency or personality that confuses adjective use. Even with our training procedures, it still seems that the raters may have coded God’s actions and characteristics in some cases simply because that is what many participants wrote about. Even in these situations, the coding procedures were useful in that understanding what God does still informs what God is.

The top-down language analysis procedures (i.e., the LIWC) added confidence to the hand-coding procedures. While the LIWC, and other automatic language analysis techniques, are not directly useful for identifying metaphors [24], they can help indirectly, especially in the current case. Participants were tasked with simply describing what they think the word “God” means. As such, for the most part, their responses only contained language that they used to serve this goal. This language can indirectly reveal the metaphoric imagery of a person’s conceptualization of God. The easiest example is the use of personal pronouns. By using “he” to describe God in an agentic manner, it *suggests* that the person thinks of God as a male human (or entity). The use of power-related words *suggests* that a person conceptualizes God as a powerful force (e.g., “creator” or “higher power”). The LIWC results enhance and support the results of our coding procedure.

Using a bottom-up or data-driven language frequency analysis procedures we identified common single words from the corpus we analyzed. Here, we opted for a word cloud. This allowed us to visualize the specific words used and to make inferences. Again, we were able to confirm the results from the coding and LIWC procedures, providing added confidence to our conclusions. For example, the words “creator” and “father” stand out in the word cloud and are easily represented in the “GOD IS POWER” and “GOD IS HUMAN/MALE” source categories. Of course, there are a lot of words that are not useful for metaphor identification and are also descriptors of God’s attributes. Furthermore, for both automated language analysis processes, we cannot determine if the words were used in negation (e.g., “God is not a human”). But, even then, negating that God is x somewhat suggests that people are using x to think about the God concept. Overall, though, our triangulation approach makes up for the limitations and difficulties of each method.

Finally, we have used a number of terms in describing our theory, procedures, and implications (e.g., “omniscient” or “omnipotent”) and labeled source categories (e.g., “power” or “human”) under which raters coded participants’ descriptions of God. We want to be clear that participants did not necessarily use these words. Most participants did not write “God is human,” for example. Therefore, our analyses were necessarily qualitative in the sense that we had to “translate” participants’ descriptions into the probable metaphoric source categories we perceived them as reflecting. As a result, different researchers may have coded some descriptors into different categories or felt that they were not metaphoric. Such issues of subjectivity are a limitation of most hand-coding procedures and their reproducibility [24, 40]. We, again, point to the triangulation approach we have adopted here as a way to reduce the influence of such limitations.

Aside from the limitations of our analysis procedures, there are additional issues to consider. Primarily, our sample was decidedly non-representative, as it consisted of mostly Christian participants in the U.S. This limits our ability to make broad conclusions or to generalize to universal God conceptualizations. Even so, some researchers suggest that religiosity and God-belief might stem from evolutionary pressures, which could explain the near-universal belief in the supernatural, the existence of creation myths, the performance of religious rituals, and the similarity of anthropomorphic Gods across religions [41]. Further, even though we had relatively small samples of non-Christian groups, there was striking consistency in the frequency of the metaphoric source categories the participants used across affiliations, for the most part. Even so, our cross-religion comparisons should be interpreted cautiously until future work can investigate metaphors for God from diverse religious and cultural samples.

## Conclusions

Attempts to understand life have plagued humans likely since the beginning of consciousness. To find meaning in life is to reduce ultimate uncertainty, a state that humans universally hope to avoid [42]. One common and, likely, entirely natural salve to the unbearable uncertainty of life’s meaning is God [41]. The concept of God, or religion, provides answers and guidance to life’s most fundamental questions and shapes people’s worldview. As such, researchers have focused on identifying how people conceptualize God. Metaphors seem perfectly apt for the task of conceptualizing God.

The current investigation represents an exploration of the most common metaphors people use to describe the God concept in a predominantly Christian sample. We identified 16 relatively common source categories that our sample used to refer to God. With these findings, future work can start to explore the consequences of individual differences in God metaphor endorsement. Indeed, understanding God in one way over another may reflect fundamental differences in how one understands the world. Metaphors for God may be one way to discern these differences.
